# Commentary: Immune System Dysregulation During Spaceflight: Potential Countermeasures for Deep Space Exploration Missions

**DOI:** 10.3389/fimmu.2018.02024

**Published:** 2018-09-04

**Authors:** Joseph J. Bevelacqua, S. M. J. Mortazavi

**Affiliations:** ^1^Bevelacqua Resources, Richland, WA, United States; ^2^Diagnostic Imaging Department, Fox Chase Cancer Center, Philadelphia, PA, United States; ^3^INIRPRC, Shiraz University of Medical Sciences, Shiraz, Iran

**Keywords:** space radiation, immune system, countermeasures, deep space missions, radioadaptation

We read with great interest the paper authored by Crucian et al. “Immune System Dysregulation During Spaceflight: Potential Countermeasures for Deep Space Exploration Missions” that is published in Frontiers in Immunology (1). The authors have addressed the key issue of dysregulation of the immune system in deep space missions and state that the immune system is strongly susceptible to a wide variety of stressors ranging from psychological to physical and local environmental factors (including exposure to oxidative stress and radiation). They also note that on deep space missions, beyond the range of the Earth's protective magnetic field (magnetosphere), factors such as ionizing radiation may increase oxidative stress and change both the rate of DNA damage and the effectiveness of the mechanisms involved in DNA repair. Oxidative stress also can be increased by DNA damage which leads to chronic inflammation and ultimately failure of the functions of the immune system. Although the paper authored by Crucian et al. is well-structured and can be considered as a significant contribution to this field, it has three important shortcomings.

The first shortcoming comes from ignoring the reports indicating that low doses of ionizing radiation [e.g., the normal levels of space radiation inside a well-shielded spacecraft when a large solar particle event (SPE) is not in progress] can reduce the oxidative damage in normal tissues. For example, Scott has previously reported that low dose radiation (LDR) stimulates antioxidant production and protects the organism from oxidative damage (2). Moreover, the authors have not considered that dysregulation of the immune system can be due to factors other than radiation. In this light, even pre-mission stress is reported to be of crucial importance (3, 4). Thus, it is likely that immune parameters are affected by pre-mission stress (5).

Second, the authors suggest that astronauts should be supplied with vitamins E, A, and C “*Astronauts need to be adequately supplied with vitamins E, A, and C. Whether provision of larger amounts of these as nutraceuticals will be beneficial for immune function warrants further investigation, on Earth and in space*.” The role of vitamin C in deep space missions is more important than reported by Crucian et al. Some evidence show that administration of vitamin C can be effective even after exposure to sparsely ionizing radiation (6, 7). Vitamin C can be introduced as a promising non-toxic, cost-effective, easily available radiation mitigator which can be used hours after a significant irradiation event (e.g., a large SPE) (6).

Finally, the key issue of radioadaptation as a factor that affects the human immune system is not considered in this paper (8–12). Radioadaptation increases the body's resistance to a high level stressor [e.g., a large SPE or exposure to intense high energy, high atomic number (HZE) particles] after a pre-exposure to a low level stressor [e.g., chronic galactic cosmic radiation] (11–14). By referring to reports about the importance of radioadaptation in deep space missions (15–18), a 2016 NASA report states it is realistic to expect that cells will be exposed to multiple hits of protons before being traversed by an HZE particle (i.e., induction of radioadaptation) (19). Besides the NASA report, a paper authored by 30 scientists from US, Canada, UK, Russia, and Belgium has also confirmed the key role of biological protection of astronauts (20). The authors state that regarding space, it is important to understand how radioadaptive responses can be induced by exposure to high LET radiation. It worth noting that a pre-exposure to low dose radiation can stimulate the defense mechanisms such as increasing antioxidant levels reducing the endogenous DNA damage, increasing DNA repair capacity, and increasing apoptosis of damaged cells (21).

In summary, the paper by Crucian et al. is an important contribution to studies of the biological effects that can occur during space missions. This commentary adds three additional considerations that should be considered when reviewing the conclusions of Crucian et al. The authors hope that this commentary will further stimulate research in studies of the effects of space radiation and enhance the health and safety of astronauts participating is space missions.

## Author contributions

SM has fully reviewed and criticized the original article, drafted the commentary, reviewed, and approved the final manuscript. JB has also reviewed and criticized the original article, assisted in drafting the commentary, reviewed and approved the final manuscript.

### Conflict of interest statement

The authors declare that the research was conducted in the absence of any commercial or financial relationships that could be construed as a potential conflict of interest.
